# Effects of single and integrated water, sanitation, handwashing, and nutrition interventions on child soil-transmitted helminth and *Giardia* infections: A cluster-randomized controlled trial in rural Kenya

**DOI:** 10.1371/journal.pmed.1002841

**Published:** 2019-06-26

**Authors:** Amy J. Pickering, Sammy M. Njenga, Lauren Steinbaum, Jenna Swarthout, Audrie Lin, Benjamin F. Arnold, Christine P. Stewart, Holly N. Dentz, MaryAnne Mureithi, Benard Chieng, Marlene Wolfe, Ryan Mahoney, Jimmy Kihara, Kendra Byrd, Gouthami Rao, Theodora Meerkerk, Priscah Cheruiyot, Marina Papaiakovou, Nils Pilotte, Steven A. Williams, John M. Colford, Clair Null

**Affiliations:** 1 Civil and Environmental Engineering, Tufts University, Medford, Massachusetts, United States of America; 2 Civil and Environmental Engineering, Stanford University, Stanford, California, United States of America; 3 Kenya Medical Research Institute, Nairobi, Kenya; 4 Innovations for Poverty Action, Kakamega, Kenya; 5 Division of Epidemiology, School of Public Health, University of California, Berkeley, California, United States of America; 6 Department of Nutrition, University of California, Davis, California, United States of America; 7 Smith College, Northampton, Massachusetts, United States of America; 8 Department of Life Sciences, Natural History Museum, London, United Kingdom; 9 Center for International Policy Research and Evaluation, Mathematica Policy Research, Washington, District of Columbia, United States of America; Umeå Centre for Global Health Research, Umeå University, SWEDEN

## Abstract

**Background:**

Helminth and protozoan infections affect more than 1 billion children globally. Improving water quality, sanitation, handwashing, and nutrition could be more sustainable control strategies for parasite infections than mass drug administration, while providing other quality of life benefits.

**Methods and findings:**

We enrolled geographic clusters of pregnant women in rural western Kenya into a cluster-randomized controlled trial (ClinicalTrials.gov NCT01704105) that tested 6 interventions: water treatment, improved sanitation, handwashing with soap, combined water treatment, sanitation, and handwashing (WSH), improved nutrition, and combined WSH and nutrition (WSHN). We assessed intervention effects on parasite infections by measuring *Ascaris lumbricoides*, *Trichuris trichiura*, hookworm, and *Giardia duodenalis* among children born to the enrolled pregnant women (index children) and their older siblings. After 2 years of intervention exposure, we collected stool specimens from 9,077 total children aged 2 to 15 years in 622 clusters, including 2,346 children in an active control group (received household visits but no interventions), 1,117 in the water treatment arm, 1,160 in the sanitation arm, 1,141 in the handwashing arm, 1,064 in the WSH arm, 1,072 in the nutrition arm, and 1,177 in the WSHN arm. In the control group, 23% of children were infected with *A*. *lumbricoides*, 1% with *T*. *trichiura*, 2% with hookworm, and 39% with *G*. *duodenalis*. The analysis included 4,928 index children (median age in years: 2) and 4,149 older siblings (median age in years: 5); study households had an average of 5 people, <10% had electricity access, and >90% had dirt floors. Compared to the control group, *Ascaris* infection prevalence was lower in the water treatment arm (prevalence ratio [PR]: 0.82 [95% CI 0.67, 1.00], *p* = 0.056), the WSH arm (PR: 0.78 [95% CI 0.63, 0.96], *p* = 0.021), and the WSHN arm (PR: 0.78 [95% CI 0.64, 0.96], *p* = 0.017). We did not observe differences in *Ascaris* infection prevalence between the control group and the arms with the individual interventions sanitation (PR: 0.89 [95% CI 0.73, 1.08], *p* = 0.228), handwashing (PR: 0.89 [95% CI 0.73, 1.09], *p* = 0.277), or nutrition (PR: 86 [95% CI 0.71, 1.05], *p* = 0.148). Integrating nutrition with WSH did not provide additional benefit. *Trichuris* and hookworm were rarely detected, resulting in imprecise effect estimates. No intervention reduced *Giardia*. Reanalysis of stool samples by quantitative polymerase chain reaction confirmed the reductions in *Ascaris* infections measured by microscopy in the WSH and WSHN groups. Trial limitations included imperfect uptake of targeted intervention behaviors, limited power to detect effects on rare parasite infections, and that it was not feasible to blind participants and sample collectors to treatment status. However, lab technicians and data analysts were blinded to treatment status. The trial was funded by the Bill & Melinda Gates Foundation and the United States Agency for International Development.

**Conclusions:**

Integration of improved water quality, sanitation, and handwashing could contribute to sustainable control strategies for *Ascaris* infections, particularly in similar settings with recent or ongoing deworming programs. Combining nutrition with WSH did not provide further benefits, and water treatment alone was similarly effective to integrated WSH. Our findings provide new evidence that drinking water should be given increased attention as a transmission pathway for *Ascaris*.

**Trial registration:**

ClinicalTrials.gov NCT01704105.

## Abstract 2

Interventions for improved water and hygiene in pregnant women in Kenya resulted in fewer Ascaris infections in newborns, Amy Pickering and colleagues reveal.

## Author summary

**Why was this study done?:**

- Intestinal worm and protozoan infections affect >1 billion children and are associated with growth faltering and impaired cognitive development.
- High reinfection rates can prevent mass drug administration programs from eliminating transmission.
- Improved water quality, sanitation, handwashing, and nutrition could interrupt environmental transmission of parasites, but few trials evaluating these interventions have measured parasite infections as an outcome.

**What did the researchers do and find?:**

- The authors conducted a randomized controlled trial among a birth cohort to test if single and combined improved drinking water quality, sanitation, handwashing, and nutrition interventions can reduce intestinal worm and *Giardia* infections. The authors measured parasite infections after 2 years of intervention exposure.
- The authors demonstrated that water treatment alone and integrated water, sanitation, and handwashing interventions can sustainably reduce roundworm (*Ascaris*) infection prevalence among young children in Kenya.
- Improved nutrition did not enhance the effectiveness of the water, sanitation, and handwashing interventions, and none of the interventions reduced *Giardia*.

**What do these findings mean?:**

- Improving water quality, sanitation, and handwashing concurrently in the household environment can protect children from infection with *Ascaris*.
- The study also provides evidence that water treatment alone may provide a similar level of protection against *Ascaris* infection, suggesting that combining water, sanitation, and handwashing interventions does not yield greater health benefits than implementing single interventions.
- Treating drinking water is a relatively unexplored strategy for controlling intestinal worm infections, and drinking water should be given increased attention as an ingestion exposure pathway for *Ascaris* eggs.

## Introduction

Intestinal soil-transmitted helminth (STH) infections, including *Ascaris lumbricoides*, *Trichuris trichiura*, and hookworm, and the protozoan *Giardia duodenalis* are common parasitic infections among children in low-resource settings and are neglected tropical diseases. Globally, STHs are estimated to affect 1.45 billion people [1], while *Giardia* has been cited as the most common enteropathogen in low-income countries [2]. STH and *Giardia* infections can result in poor absorption of nutrients and weight loss [3,4]. There is some evidence that STH and *Giardia* infections, even when asymptomatic, may contribute to growth faltering and impaired cognitive development [5–8]. Longitudinal cohort studies in Bangladesh and Brazil have identified early infection with *Giardia* as a risk factor for stunting among children [7,9]. In Peru, children with multiple *Giardia* infections per year during the first 2 years of life had lower cognitive function scores at age 9 years than children with 1 or fewer *Giardia* infections [10]. Evidence on the effect of child STH infections on child growth, cognitive development, and school performance has been mixed and strongly debated by experts, with some suggesting additional evidence is needed [5,6,11–14].

School-based targeted mass drug administration (MDA) campaigns have been the cornerstone of the global strategy to control STH infections; however, high reinfection rates limit the ability of MDA to achieve sustained reduction in STH infection prevalence [15]. *Ascaris*, *Trichuris*, *Giardia*, and *Ancylostoma duodenale* are primarily transmitted through the fecal—oral ingestion route, although *A*. *duodenale* as well as *Necator americanus* can be transmitted transdermally [3]. A meta-analysis of studies from settings with medium-to-high endemic STH prevalence identified an average reinfection rate for *Ascaris* at 12 months of 94% of baseline prevalence, while the average 12-month reinfection rates for *Trichuris* and hookworm were 82% and 57%, respectively [16]. To achieve elimination of STH transmission, it has been suggested that MDA control efforts may need to be integrated with improved water, sanitation, and handwashing [17]. Control of *Giardia* has historically relied on drug treatment after diagnosis as well as exposure prevention by water treatment and improved sanitation, but zoonotic transmission can complicate exposure prevention: Few interventions have been developed to prevent human exposure to animal fecal contamination [18].

Recent systematic reviews suggest that improved water, sanitation, and handwashing can reduce the odds of STH and *Giardia* infections, though the quality of the evidence base remains poor and consists almost exclusively of observational analyses [2,19]. Two randomized controlled trials (RCTs) in rural India found no impact of community sanitation interventions on helminth infections; however, both studies reported low usage rates of toilets among intervention households [20,21]. A recent cluster-randomized trial in Timor-Leste found no additional benefit from combining improved water, sanitation, and handwashing with deworming over deworming alone [22]. We were able to identify 3 previous RCTs evaluating water and sanitation effects on *Giardia* [2]. Two water treatment trials in Guatemala and Rwanda with small sample sizes (*n* < 200 participants per arm) did not detect an effect on serological measures of *Giardia* [23,24], while a community-level sanitation trial detected a reduction in *Giardia* infection prevalence in rural India [20].

An individual’s susceptibility to STH and *Giardia* infection is influenced by exposure and immune response. A recent systematic review concluded that there was some evidence that nutritional supplementation decreases the risk of infection or reinfection with STHs, but studies have been of low quality [25]. Plausible mechanisms by which nutrition might reduce STH or *Giardia* infection are through improvements in effective immune response, including repair of cell damage caused by parasite infection, and through changes to the gut microbiome [26,27].

We conducted a cluster-randomized controlled trial (WASH Benefits) in rural Kenya to assess the effects of water, sanitation, handwashing, and nutrition interventions delivered alone and in combination on STH and *Giardia* infections among a birth cohort. STH and *Giardia* infections were prespecified as trial outcomes before the trial began [28]. In a separate paper, we reported the effects of the interventions on child growth and diarrhea [29]. The trial’s nutrition intervention was the only component that improved child growth, and none of the interventions reduced diarrhea [29]. Here, we report intervention effects on *Ascaris*, *Trichuris*, hookworm, and *Giardia* infections measured after 2 years of intervention exposure.

## Methods

### Study design

The trial protocol and detailed methods are published [28]. The trial was registered at ClinicalTrials.gov, identification number: NCT01704105. The study protocol was approved by the Committee for Protection of Human Subjects at the University of California, Berkeley (protocol number 2011-09-3654), the Institutional Review Board at Stanford University (IRB-23310), and the Scientific and Ethics Review Unit at the Kenya Medical Research Institute (protocol number SSC-2271). Innovations for Poverty Action enrolled participants, implemented the intervention delivery, and collected the data. Mothers provided written informed consent for themselves and their children.

**Fig 1:**
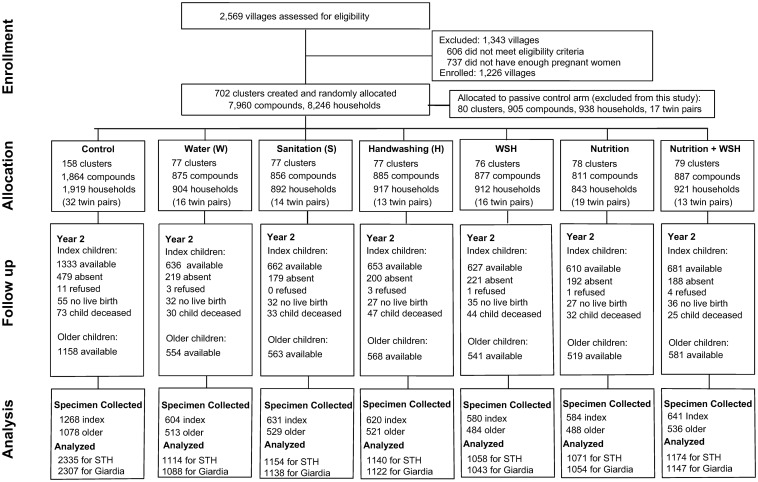
Trial profile and participant flow. STH, soil-transmitted helminth.

Clusters of eligible pregnant women were randomized by geographic proximal blocks into 1 of 8 study arms: water treatment (chlorine treatment of drinking water); improved sanitation(provision of toilets with plastic slabs and hardware to manage child feces); handwashing with soap; combined water treatment, sanitation, and handwashing (WSH); improved nutrition (infant and young child feeding counseling plus small-quantity lipid-based nutrient supplements [LNSs]); combined WSH and nutrition (WSHN); a double-sized active control; and a passive control. The trial included a passive control arm to test if promoter visits alone (active control) had an effect on the trial’s primary outcomes diarrhea and growth; children in the passive control arm were purposively excluded from parasitology measurement (Fig 1).

We conducted a cluster-randomized trial because there could have been behavior and infectious disease interactions between neighboring households. Villages were eligible for selection into the study if they were rural, the majority of the population lacked access to piped water supplies, and there were no other ongoing WSH or nutrition programs. Within selected villages, a census was conducted to identify eligible pregnant women in their second or third trimester who planned to continue to live at their current residence for the next year. Since interventions were designed to reduce child exposure to pathogens through a cleaner environment and exclusive breastfeeding, we enrolled pregnant women to allow time for intervention delivery to occur prior to or as close to birth as possible. After the census, clusters were formed from 1–3 neighboring villages and had a minimum of 6 pregnant women per cluster after the enrollment survey (each village could only be assigned to 1 cluster). Enrolled study compounds were thus a small proportion of the total number of compounds residing in each cluster. Children born to enrolled pregnant women were considered “index” children. We measured parasite infections approximately 27 months post-enrollment (which equates to a minimum of 24 months of intervention exposure since intervention hardware was delivered <3 months after enrollment). Outcomes were assessed among index children, including twins, as well as among 1 older child in the index child’s compound to understand the effect of the interventions on both preschool-aged and school-aged children. The older child was selected by enrolling the youngest available child within the age range of 3–15 years old, with priority for a sibling in the index child’s household.

### Baseline survey

A survey at enrollment measured household socioeconomic characteristics and demographics (including maternal age, maternal education, electricity access, type of floor, and number of people in the household), as well as water, sanitation, and handwashing infrastructure and behaviors (including type of water source, reported water treatment, defecation location, type of toilet, and presence of water and soap at a handwashing station). In addition, at study enrollment we measured *Giardia*, *Entamoeba histolytica*, and *Cryptosporidium* spp. among children residing in study compounds between 18 and 27 months of age (the projected age range for index children at the end of the study) to assess baseline prevalence of these pathogens. STHs were not measured at enrollment among these proxy children because it was not logistically feasible to deworm infected children at baseline. We also collected 100-ml samples from primary drinking water sources accessed by study households and household stored drinking water (if available). We transported the samples on ice to field labs and enumerated *Escherichia coli* in each sample by membrane filtration followed by culture on MI medium.

### Randomization and blinding

A few weeks after enrollment, clusters were randomly assigned to intervention/control arms at the University of California, Berkeley, by an investigator independent of the field research team (BFA) using a random number generator. Groups of 9 geographically adjacent clusters were block-randomized into the 6 intervention arms, the double-sized active control arm, and the passive control arm (the passive control arm was not included in the parasite assessment). Participants and other community members were informed of their intervention/control group assignment after the baseline survey. Blinding (masking) of participants was not possible given the nature of the interventions. Data and stool sample collectors were not informed of cluster assignment, but could have inferred treatment status by observing intervention hardware. Lab technicians were blinded to intervention status. Two authors (AJP and JS) independently replicated the statistical analyses while blinded to intervention status.

### Intervention delivery

Intervention delivery occurred within 3 months after enrollment. In the water intervention arms (water treatment, WSH, and WSHN), community health promoters encouraged drinking water treatment with chlorine (liquid sodium hypochlorite) using either manual dispensers installed at the point of collection (community water source) in study villages or bottled chlorine provided directly to households every 6 months. In the sanitation intervention arms (sanitation, WSH, and WSHN), households in study compounds received new latrines, or existing latrines were upgraded and improved by installing a plastic slab that included a lid. All households in sanitation arm study compounds were provided with a child potty for each child <3 years as well as a “sani-scoop” to remove animal and human feces from the compound. Households were encouraged to use latrines for defecation and for disposal of child and animal feces. In the handwashing intervention arms (handwashing, WSH, and WSHN), study compounds were provided with 2 handwashing stations—near the latrine for handwashing after defecation and near the cooking area for handwashing before preparing food. Stations included dual foot-pedal-operated jerry cans that could be tipped to dispense either soapy water or rinse water. Households were responsible for keeping the stations stocked with rinse water, and community health promoters refilled soap regularly. In the nutrition intervention arms (nutrition and WSHN), small-quantity LNSs were provided to children 6–24 months of age. Children received monthly rations of LNSs for addition to their other food twice per day. Nutrition messaging included promoting dietary diversity during pregnancy and lactation, early initiation of breastfeeding, exclusive breastfeeding at age 0–6 months, continued breastfeeding through age 24 months, timely introduction of complementary foods, dietary diversity for child feeding, and child feeding during illness. Intervention delivery was at the cluster level for the water intervention (all compounds in villages assigned to the cluster had access to the chlorine dispensers), at the compound level for sanitation and handwashing (non-study compounds in the cluster did not receive handwashing stations or improved toilets), and at the child level for the nutrition intervention (only index children and their siblings under 24 months received LNSs).

Community health promoters were nominated by mothers in the community and trained to provide intervention-specific behavior change activities and instructions on hardware use and provision of nutrition supplements. They were also trained to measure the mid-upper arm circumference of the index children to identify and provide referrals for potential cases of severe acute malnutrition. Each intervention consisted of a comprehensive behavior change package of key messages; visual aids in the form of flip charts, posters, and reminder cue cards; interactive activities with songs, games, or pledges to commit to practice target behaviors; and the distribution of arm-specific hardware, products, or supplements. Households in the active control group received visits from promoters to measure child mid-upper arm circumference and provide malnutrition referrals, but did not receive any intervention-related hardware or messaging. Promoters were instructed to visit households monthly. Key messages and promoter materials are available at https://osf.io/fs23x/.

Adherence to the interventions was measured during unannounced household visits after 1 year and 2 years of intervention exposure (S1 Text).

### Measurement of parasite infections

Stool samples were collected from index children and older children in sterile containers and transported on ice to the closer of 2 central field labs located in Kakamega and Bungoma. Field staff revisited households up to 3 times to collect stool samples. *A*. *lumbricoides*, *T*. *trichiura*, and hookworm eggs were immediately enumerated (same day) by double-slide Kato—Katz microscopy with 41.7-mg templates. Both slides created from each stool sample were counted by a trained parasitologist, and 2 different parasitologists counted each slide from the same sample. A supervisor with expertise in STH egg identification reviewed 10% of all slides, and any discrepancies were corrected. STH egg counts were averaged for analysis if both slides from 1 stool sample were positive; if 1 slide was negative, the count for the positive slide was used for analysis. Two aliquots of stool (1 mixed with ethanol) were transported on dry ice to the Eastern and Southern Africa Centre of International Parasite Control laboratory at the Kenya Medical Research Institute in Nairobi, Kenya, for further analysis.

One aliquot was analyzed by monoclonal enzyme-linked immunosorbent assay (ELISA) (*Giardia II*, Alere International, Galway, Ireland) for the presence or absence of *G*. *duodenalis* cysts. Samples were measured by ELISA in duplicate; if there was a discrepancy between duplicates, the sample was rerun. DNA was extracted from the other aliquot (preserved in ethanol) for stool samples collected from children in the control, WSH, and WSHN groups. Four quantitative polymerase chain reaction (qPCR) assays were run in duplicate on each sample to detect the following targets: *N*. *americanus*, *A*. *duodenale*, *T*. *trichiura*, and *A*. *lumbricoides* (see S1 Text for further details) [30].

### Outcomes

STH and *Giardia* infections were prespecified outcomes in the parent WASH Benefits trial prior to the start of data collection; see Fig 3 in Arnold et al. [28]. Parasite infections were measured after 2 years of intervention exposure. The main indicators of parasite infections were the prevalence of each individual STH infection, any STH infection, and *Giardia* infection among index and older children from the same compound. Additional indicators of parasite infections included intensity of *Ascaris*, *Trichuris*, and hookworm measured in eggs per gram (epg) of feces; intensity binary category of *Ascaris* infection, measured as low intensity (1–5,000 epg) or moderate/high intensity (>5,000 epg), following World Health Organization (WHO) cutoffs; prevalence of coinfection with 2 or 3 STHs; and prevalence of coinfection with *Giardia* and any STH. The trial’s original protocol included *E*. *histolytica* and *Cryptosporidium* spp. as additional protozoan endpoints. At enrollment, *Giardia* prevalence was 40% among 535 children 18–27 months old in study compounds, while *Cryptosporidium* spp. prevalence was 1% and *E*. *histolytica* prevalence was 0%. We determined that the extremely low baseline prevalence of *E*. *histolytica* and *Cryptosporidium* spp. made these trial endpoints futile due to limited statistical power, and since each required a separate assay on the ELISA platform, the study’s steering committee decided to not test for them at follow-up.

### Sample size calculations

All households in all clusters enrolled into the main trial were invited to participate in the measurement of parasite infections. The main trial was powered for a minimum detectable effect of 0.15 in length-for-age *Z* score and a relative risk of diarrhea of 0.7 or smaller for a comparison of any intervention with the double-sized control group, assuming a type I error (α) of 0.05 and power (1 − β) of 0.8, 10% loss to follow-up, and a 1-sided test for a 2-sample comparison of means (the main trial statistical analysis plan was later changed to employ 2-sided tests). This led to a planned design of 100 clusters per arm and 10 index children per cluster. Given this design and a single post-intervention measure, we estimated that the trial’s sample size would be sufficient at 80% power with a 2-sided α of 0.05 to detect a relative reduction of 18% in infection prevalence of any parasite (2-sided tests were planned due to a lack of evidence that all interventions would have a protective effect). Our minimum detectable effect calculations assumed 50% prevalence in the control arm, a village intraclass correlation (ICC) of 0.14, 2 children measured per enrolled household (index child plus an older sibling), and 70% successful stool collection and analysis. For perspective, this minimum detectable effect is much smaller than typical effect sizes reported in meta-analyses of the association between improved water, sanitation, and handwashing and helminth/protozoan infections (e.g., odds ratios between 0.46 and 0.58 for sanitation facilities and helminth infections) [2,31].

### Statistical analysis

All statistical analyses and comparisons between arms (water treatment, sanitation, handwashing, WSH, nutrition, and WSHN compared to active control) were prespecified prior to unblinding of investigators, and the analysis plan was published with a time stamp on the Open Science Framework (https://osf.io/k2s47/). Replication scripts and data are also provided at the same link. Our alternative hypothesis for all comparisons was that group means were not equal (2-sided tests). We estimated unadjusted and adjusted intention-to-treat effect differences between study arms using targeted maximum likelihood estimation with influence-curve-based standard errors that treated clusters as independent units and allowed for outcome correlation within clusters [32,33]. Our parameters of interest for dichotomous outcomes were prevalence ratios (PRs) (prevalence in the intervention group divided by the prevalence in the control group). Our parameter of interest for helminth intensity was the relative fecal egg count reduction. We calculated the relative reduction using both geometric and arithmetic means. We did not perform statistical adjustments for multiple outcomes to preserve interpretation of effects and because many of our outcomes were correlated [34]. We estimated adjusted parameters by including variables that were associated with the outcome, to potentially improve the precision of our estimates. We prescreened covariates (S1 Text) to assess whether they were associated (*p*-value < 0.2) with each outcome prior to including them in adjusted statistical models [35]. We conducted subgroup analyses to explore effect modification on *Ascaris* and *Giardia* infection presence by the following factors: index child status (LNSs were given only to index children and siblings under 24 months), consumed deworming medicine in past 6 months (*Ascari*s only), consumed soil in past week (index children only), >8 people in compound, and time since defecation before stool collection. Statistical analyses were conducted using R version 3.3.2 (https://www.r-project.org).

## Results

### Enrollment

Pregnant women were enrolled into the cluster-randomized controlled trial from Kakamega, Bungoma, and Vihiga counties in Kenya’s western region. Enrollment occurred between November 27, 2012, and May 21, 2014; 8,246 pregnant women were enrolled. Clusters with an average of 12 eligible pregnant women each were randomized by geographic proximal blocks into 1 of 8 study arms: water treatment (chlorine treatment of drinking water); improved sanitation (provision of toilets with plastic slabs and hardware to manage child feces); handwashing with soap; combined WSH; improved nutrition (infant and young child feeding counseling plus small-quantity LNSs); combined WSHN; a double-sized active control; and a passive control. Children in the passive control arm were purposively excluded from parasitology measurement: Only the active control group is considered hereafter (Fig 1). Parasite infections were measured among children born to enrolled pregnant women (index children) as well as their older siblings or an older child in the same compound.

Enrollment characteristics of the study population were similar between arms (Table 1). Most households accessed springs or wells as their primary drinking water source. In the control group, 24% of households accessed unprotected water sources, such as springs, dug wells, and surface water. The microbial quality of drinking water was very poor, as has been reported previously for this study area [36]; 96% (*n* = 1,829) of source water samples and 94% (*n* = 5959) of stored drinking water samples contained *E*. *coli* contamination. Most (82%) households owned a latrine, but only 15% had access to a latrine with a slab or ventilation pipe (Table 1). Soap and water availability for handwashing at a designated handwashing location was low (<10%).

**Table 1:** Baseline characteristics by treatment assignment.

Characteristic	Control (*n* = 1,916)	Water (*n* = 903)	Sanitation (*n* = 886)	Handwashing (*n* = 911)	WSH (*n* = 909)	Nutrition (*n* = 840)	Nutrition + WSH (*n* = 920)
**Maternal**							
Age (years)	26	26	26	26	26	26	26
Completed primary school	47.9	49.4	48.4	43.8	47.1	48.6	47.7
**Paternal**							
Completed primary school	62.4	63.9	58.7	59.0	61.5	63.5	62.5
Works in agriculture	41.1	44.2	42.6	42.1	43.2	43.6	42.8
**Household**							
Number of persons	5	5	5	5	5	5	5
Has electricity	6.4	6.7	8.1	7.2	7.0	6.9	7.3
Has a cement floor	5.6	8.1	5.5	4.5	5.4	5.7	6.1
**Drinking water**							
Primary source protected	75.7	75.3	75.9	77.5	68.6	71.5	76.1
Stored water observed at home	81.5	81.3	82.0	82.8	79.5	81.0	81.0
Reported treating currently stored water	12.6	11.1	12.8	12.6	13.2	11.6	14.3
**Sanitation**							
Daily defecating in the open							
Children: 3 to <8 years	11.7	12.6	12.7	13.8	12.8	14.4	12.3
Children: 0 to <3 years	77.5	80.2	74.8	76.2	76.4	78.5	78.0
Latrine							
Owned by compound	81.7	83.2	81.2	82.8	82.7	83.1	83.5
Has slab or ventilation pipe	17.3	17.7	15.7	18.4	17.5	15.0	16.4
Visible feces on slab or floor	47.6	78.3	76.3	50.1	52.5	50.4	50.2
Has a child potty	2.3	2.7	2.0	3.1	2.6	1.7	2.2
Human feces observed in the compound	8.6	7.3	8.0	9.3	8.1	8.7	9.5
**Handwashing**							
Handwashing station has water and soap	5.0	6.2	4.7	5.6	6.9	6.7	5.7

Data are mean or percent. Protected water sources include piped water, borewells, protected springs, protected dug wells, and rainwater collection.

WSH, water treatment, sanitation, and handwashing.

### Indicators of intervention uptake

After 1 year of intervention, 89%–90% of households that received the sanitation intervention had access to an improved latrine with a slab or a ventilation pipe (compared to 18% in the control arm), and 79%–82% of these had access to an improved latrine after 2 years of intervention. In the water intervention arms, 40%–44% of households had a detectable chlorine residual in their stored drinking water at the 1-year follow-up (compared to 3% of control households), and 19%–23% had chlorine detected after 2 years. In the handwashing intervention arms, 76%–78% of households had soap and water available at a handwashing station (compared to 12% in the control arm) after 1 year, and this decreased to 19%–23% at 2 years. Consumption of LNS sachets by children in the nutrition intervention arms was 95%–96% of the expected 2 sachets per day at the 1-year follow-up, and 114%–116% of expected at the 2-year follow-up (>100% is possible because additional LNS sachets were delivered in case of future delivery delays) (S1 and S2 Tables).

### Infection prevalence

STH and *Giardia* infections were measured after 2 years of exposure to the interventions. We collected stool specimens from 9,077 children aged 2–15 years at the 2-year survey during January 2015–July 2016, including 4,928 index children (median age in years: 2.0; IQR 1.9, 2.1) and 4,149 older children (median age in years: 5.0; IQR 4.2, 6.4) residing in an index child’s compound (Fig 1). A total of 2,346 children in 158 control clusters, 1,117 children in 77 water clusters, 1,160 children in 77 sanitation clusters, 1,141 children in 77 handwashing clusters, 1,064 children in 76 WSH clusters, 1,072 children in 78 nutrition clusters, and 1,177 children in 79 WSHN clusters provided stool specimens. Stool specimens were successfully collected from 95% (4,928 of 5,202) of available index children and from 93% (4,149 of 4,484) of available older children 2 years after intervention delivery (Fig 1 shows number of children not available due to there being no live birth, death, refusal, or absence; S3 and S4 Tables show characteristics of children lost to follow-up, by treatment status). In the control group, 22.6% of children were infected with *Ascaris* (ICC: 0.10), 2.2% with hookworm (ICC: 0.04), 1.2% with *Trichuris* (ICC: 0.07) (measured by Kato–Katz microscopy), and 39% with *Giardia* (measured by ELISA) (S5 Table). *Ascaris* infection prevalence was similar for index children (22.8%) and older children (22.3%) in the control group. Caregivers reported that 39% of index children and 10% of older children had consumed soil in the past 7 days.

### Effect of interventions on parasite infection prevalence

**Fig 2:**
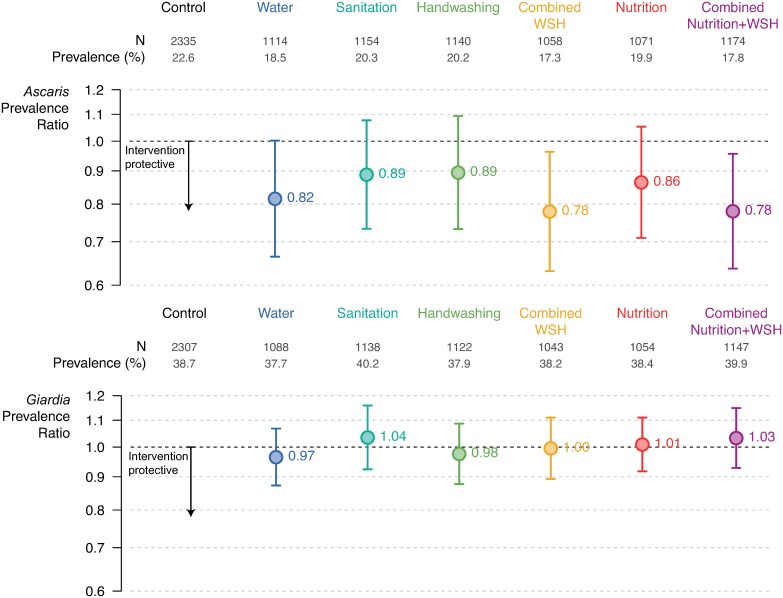
Effect of the interventions on infection with *Ascaris* and *Giardia*: Data includes all index children and older siblings combined. Prevalence ratios estimated by targeted maximum likelihood estimation. Error bars show 95% confidence intervals for the prevalence ratios. WSH, water treatment, sanitation, and handwashing.

Infection prevalence of each STH, any STH, and *Giardia* was compared between each intervention group (water treatment, sanitation, handwashing, WSH, nutrition, and WSHN) and the double-sized active control group; see Methods for further details of the analysis. Compared to the control group, *Ascaris* infection prevalence was 18% lower in the water arm (PR: 0.82 [95% CI 0.67, 1.00]), 22% lower in the combined WSH arm (PR: 0.78 [95% CI 0.63, 0.96]), and 22% lower in the WSHN arm (PR: 0.78 [95% CI 0.64, 0.96]) (Fig 2; S5 Table). We did not observe that the individual interventions sanitation (PR: 0.89 [95% CI 0.73, 1.08]), handwashing (PR: 0.89 [95% CI 0.73, 1.09]), or nutrition (PR: 86 [95% CI 0.71, 1.05] reduced *Ascaris* infection on their own (Fig 2). The combined WSH intervention reduced infection with any STH by 23% (PR: 0.77 [95% CI 0.63, 0.95]), and the combined WSHN intervention reduced infection with any STH by 19% (PR: 0.81 [95% CI 0.66, 0.98]) (S5 Table). No interventions significantly reduced the prevalence of hookworm and *Trichuris*, though the low prevalence in the control arm meant that any reduction due to intervention would be difficult to detect in the trial (S5 Table). No interventions reduced *Giardia* prevalence (Fig 2).

**Fig 3:**
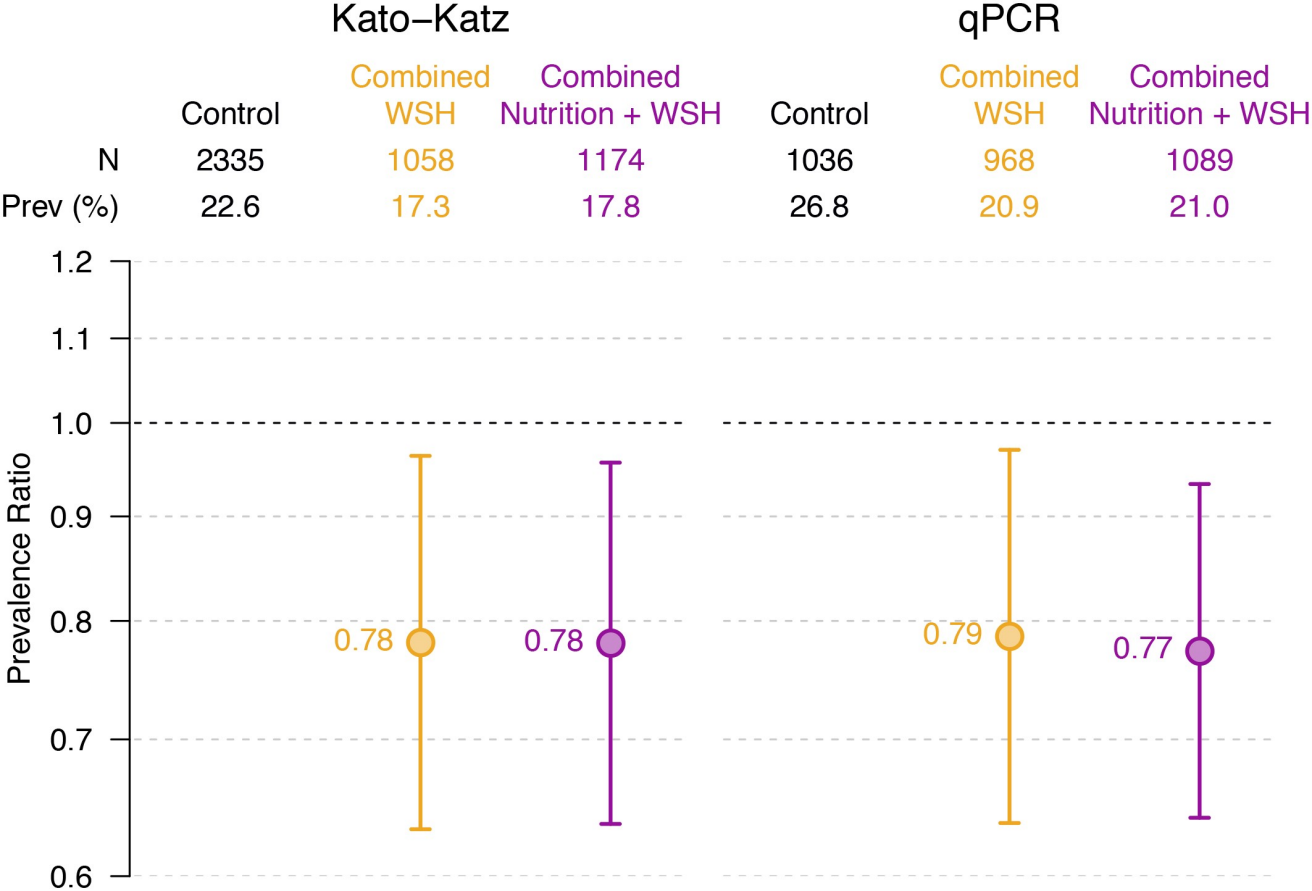
Effect of the combined interventions on infection with *Ascaris* estimated with Kato–Katz microscopy (left) and by qPCR (right). Prevalence ratios estimated by targeted maximum likelihood estimation. Error bars show 95% confidence intervals for the prevalence ratios. qPCR, quantitative PCR; WSH, water treatment, sanitation, and handwashing.

We reanalyzed all stool samples collected from children enrolled in the control, WSH, and WSHN arms by qPCR to validate our estimates based on microscopy measurements. These 3 arms were selected for the qPCR subset analysis prior to unblinding of investigators to results and were chosen based on the hypothesis that these arms would be the most likely to have low-intensity STH infections if any of the interventions were effective. qPCR analyses resulted in almost identical intervention effect estimates to those based on microscopy (Fig 3; S6 Table). Compared to the control group, *Ascaris* infection prevalence was 21% lower (PR: 0.79 [95% CI 0.64, 0.97]) in the WSH group and 23% lower (PR: 0.77 [95% CI 0.64, 0.93]) in the WSHN group. We also did not detect any significant effects of the interventions on *Trichuris* or hookworm infections using qPCR data (S6 Table).

### Effect of interventions on infection intensity

*Ascaris* infection intensity was lower in children in the water arm (fecal egg count reduction [FECR] with geometric means: −16% [95% CI −32%, −1%]), the WSH arm (FECR: −19% [95% CI −33%, −5%]), and the WSHN arm (FECR: −18% [95% CI −32%, −4%]) compared to the control arm; FECR with arithmetic means showed similar results (Table 2). The prevalence of heavy/moderate intensity *Ascaris* infections was 10.0% in the water arm, 10.9% in WSH, and 10.3% in WSHN compared to 12.7% in the control arm; these differences were not statistically significant at the 95% confidence level (S5 Table).

**Table 2:** Effect of the interventions on infection intensity, measured by FECR with arithmetic and geometric means.

Outcome and arm	*N*	Geometric mean				Arithmetic mean			
		Log10 mean*, epg	FECR	95% CI	*p*-Value	Arithmetic mean, epg	FECR	95% CI	*p*-Value
***Ascaris* FECR**									
Control	2,335	0.60				3,641			
Water	1,114	0.40	−0.16	−0.32, −0.01	0.04	2,682	−0.26	−0.52, −0.01	0.04
Sanitation	1,154	0.50	−0.09	−0.25, 0.07	0.27	3,443	−0.04	−0.32, 0.23	0.75
Handwashing	1,140	0.50	−0.08	−0.25, 0.08	0.31	3,386	−0.03	−0.34, 0.28	0.85
WSH	1,058	0.40	−0.19	−0.33, −0.05	0.01	2,571	−0.27	−0.52, −0.02	0.03
Nutrition	1,071	0.50	−0.10	−0.25, 0.04	0.16	3,303	−0.11	−0.34, 0.12	0.35
Nutrition + WSH	1,174	0.40	−0.18	−0.32, −0.04	0.01	2,927	−0.21	−0.46, 0.03	0.09
**Hookworm FECR**									
Control	2,335	−0.25				12			
Water	1,114	−0.23	0.02	−0.02, 0.05	0.37	10	−0.20	−0.84, 0.44	0.54
Sanitation	1,154	−0.24	0.01	−0.02, 0.04	0.42	10	−0.16	−0.90, 0.57	0.67
Handwashing	1,140	−0.21	0.03	0.00, 0.07	0.08	23	0.93	−1.39, 3.25	0.43
WSH	1,058	−0.26	−0.02	−0.04, 0.01	0.18	3	−0.74	−0.91, −0.58	0.00
Nutrition	1,071	−0.23	0.03	−0.01, 0.06	0.14	12	0.16	−1.17, 1.50	0.81
Nutrition + WSH	1,174	−0.23	0.02	−0.01, 0.06	0.22	24	1.02	−1.87, 3.91	0.49
***Trichuris* FECR**									
Control	2,335	−0.27				6			
Water	1,114	−0.27	0.00	−0.03, 0.03	0.92	6	0.04	−1.91, 1.98	0.97
Sanitation	1,154	−0.27	0.00	−0.03, 0.02	0.77	4	−0.19	−1.49, 1.11	0.78
Handwashing	1,140	−0.26	0.01	−0.02, 0.04	0.46	6	0.03	−1.40, 1.46	0.97
WSH	1,058	−0.29	−0.02	−0.04, 0.00	0.06	0	−0.91	−1.07, −0.75	0.00
Nutrition	1,071	−0.28	−0.02	−0.04, 0.01	0.18	2	−0.64	−1.22, −0.05	0.03
Nutrition + WSH	1,174	−0.29	−0.02	−0.05, 0.00	0.10	1	−0.81	−1.15, −0.47	0.00

FECR estimated by targeted maximum likelihood estimation. FECRs are expressed as proportions (percentage change/100).

*Value of 0.5 epg substituted for samples below the detection limit, to calculate log-transformed mean.

epg, eggs per gram; FECR, fecal egg count reduction; WSH, water treatment, sanitation, and handwashing.

The FECR with arithmetic means indicated that children in the WSH arm had lower intensity infections with hookworm (3 epg versus 11 epg in control arm) (Table 2). In addition, the FECR with arithmetic means indicated lower *Trichuris* infection intensity in the WSH (0 epg versus 6 epg in control arm), nutrition (2 epg), and WSHN (1 epg) arms. Children who received the WSHN intervention had 27% lower prevalence of coinfection with STH and *Giardia* compared to the control group (PR: 0.73 [95% CI 0.56, 0.97]) (S4 Table). STH coinfection was rare: <2% in the control arm and at similarly low levels in intervention arms (S5 Table).

### Adjusted models and subgroup analyses

Adjusted effect estimates were similar to unadjusted effects (S5 Table). Subgroup analyses of intervention effects stratified by index children versus older children, reported soil consumption (index children only), number of people living in the compound, deworming (*Ascaris* only), and time since defecation did not show any strong effect modification (S8 Table).

## Discussion

Our findings demonstrate that an integrated water, sanitation, and handwashing intervention targeting the household environment in rural Kenya reduced *Ascaris* infection prevalence by 22%, while a water treatment intervention reduced *Ascaris* infection by 18%. Almost identical effect estimates generated by analyzing stool samples with microscopy and qPCR in a subset of arms lent additional credibility to the overall results (Fig 3). In addition, we found that improved nutrition did not enhance the effectiveness of the WSH intervention. *Trichuris* and hookworm prevalence were too low to precisely assess intervention impact in this setting, and *Giardia* was unaffected by the interventions. Although the integrated WSH intervention did not succeed in improving child growth or reducing symptomatic diarrhea in this trial [29], our findings confirm that WSH can effectively reduce helminth infection prevalence.

A limited number of RCTs have previously analyzed the effect of WSH interventions on STH infection. Several school-based RCTs combining deworming with handwashing promotion have reported significant reductions in *Ascaris* reinfection prevalence in China, Ethiopia, and Peru [37–39]. A school-based integrated WSH intervention combined with deworming in rural Kenya also reduced the odds of *Ascaris* reinfection [40]. While previous RCTs demonstrate the success of school-based deworming combined with hygiene promotion, our results contribute new evidence from a large cluster-randomized trial that improving WSH in the household environment can reduce *Ascaris* infections in a rural, low-income setting.

We did not detect an effect of the sanitation intervention alone on STH infection prevalence. One potential explanation for the lack of impact may be that transitioning households from using traditional pit latrines to pit latrines with slabs may not have a measurable impact on STH transmission. A shift from households practicing open defecation to using latrines might be more likely to reduce STH transmission, with little additional benefit from improving latrine quality. A recent trial in Côte d’Ivoire reported greater reduction in hookworm infection prevalence among communities that received a community-led total sanitation intervention (designed to reduce open defecation levels) integrated with community-wide MDA compared to community-wide MDA alone, although it should be noted that the trial was not randomized and had limited statistical power [41]. A second explanation for the lack of effect of the sanitation intervention may be that sanitation interventions are more effective at interrupting environmental transmission of pathogens when they are implemented at the community level [42], whereas our intervention only improved sanitation access in compounds with enrolled pregnant women. However, a recent cluster-randomized trial of a community-wide sanitation intervention integrated with deworming in Timor-Leste found that the intervention did not reduce helminth infection prevalence more than deworming alone [22]. A third explanation is that it could require >2 years of improved sanitation access to substantially reduce levels of helminth eggs in soil (*Ascaris* eggs can survive in soil for several years).

The reductions in *Ascaris* prevalence in the combined arms could have resulted from improved water quality alone; *Ascaris* prevalence was 18% lower in the water treatment arm than the control arm, a similar magnitude to the 22% reduction in the integrated intervention arms. Near identical reductions in *Ascaris* infection across all 3 water intervention arms suggests that water could have been an important transmission pathway in this population, which was interrupted by chlorine treatment. STH transmission through water is consistent with a recently published substudy among the control, sanitation, and WSH arms in our trial that found no effect of the sanitation intervention on STH egg prevalence in soil collected from the household entrance [43]. However, we cannot completely rule out contribution to reductions from other interventions in the combined arms; *Ascaris* prevalence was lower (20%) in the single intervention arms sanitation, handwashing, and nutrition, compared to 23% prevalence in the control arm. Chlorine is not known to inactivate *Ascaris* eggs, but 1 experimental study did find that chlorine can delay egg development and infectivity [44]; it’s possible that delayed egg infectivity could reduce the risk of consuming an infective egg through drinking water. The proportion of households using jerry cans (a plastic water container with a narrow capped opening) to safely store drinking water was slightly higher in the water intervention arms than the other arms (S1 and S2 Tables). Our findings indicate that drinking water is an understudied transmission pathway for *Ascaris*. We believe drinking water treatment should be further investigated as an STH control strategy, and that chlorine should be further explored as a method for inhibiting *Ascaris* egg development in drinking water supplies. While improved sanitation and handwashing have been suggested as control strategies for *Ascaris*, water treatment has not been previously recommended as an *Ascaris* control strategy, yet it appeared to be the most effective environmental intervention that we tested in this trial. Our results have the potential to shape future guidance for STH control programs to emphasize water treatment for *Ascaris* control.

The combined WSHN intervention was similarly effective to WSH in reducing *Ascaris* prevalence, and improved nutrition did not reduce STH or *Giardia* infection on its own. Together, these results suggest that the improved nutrition intervention did not reduce parasite infection in this population. Trials investigating the impact of micronutrient supplementation on STH infection or reinfection have reported mixed results [25]. Our results are consistent with a Kenyan trial that found no effect of school-based micronutrient supplementation on reinfection with *Ascaris* [45]. Considering that interventions in this trial did not include treatment with antiparasitic drugs, further research would be valuable to understand if LNSs could prevent parasite infections after drug treatment.

Our findings suggest that combined interventions may not achieve additive or multiplicative effects on *Ascaris* infection. Similar reductions in *Ascaris* infection prevalence were observed in the water and combined WSH arms, and in the WSH and WSHN arms. Given limited resources, combining the interventions implemented in this trial may not be a cost-effective strategy to reduce helminth infections as drinking water interventions alone may yield similar benefits.

*Giardia* prevalence was unaffected by any of the interventions in this trial. Our results stand in contrast to results from the parallel WASH Benefits trial conducted in Bangladesh [46], which detected reductions in *Giardia* infection prevalence in the handwashing, sanitation, combined WSH, and combined WSHN arms [47]. One potential explanation for the lack of intervention effects in this trial is that water could be the primary transmission pathway for *Giardia* in this study setting, and *Giardia* is highly resistant to chlorination. The majority of households in the WASH Benefits Bangladesh trial accessed protected tubewells, providing water with lower levels of fecal contamination compared to the springs and shallow wells accessed by households in this trial [36,48]. Another potential explanation is that handwashing rates with soap were not high enough at the time of measurement to interrupt *Giardia* transmission; presence of soap and water at a handwashing station decreased from 78% at 1 year to 19% at 2 years among households in the WSH arm (S1 and S2 Tables). *Giardia* is also zoonotic [4]; exposure to avian and ruminant fecal contamination in the household environment could mitigate the effect of improved sanitation on transmission. Animal feces management was not a targeted behavior of the intervention packages.

This trial had some limitations. Chlorination does not inactivate protozoa, but was selected as the most appropriate water treatment intervention for the study context considering previous local acceptability, affordability, and effectiveness against bacterial and viral enteric pathogens. We measured parasite infections 2 years after intervention delivery; measurement among the study population at 1 year could have produced different results because of higher intervention adherence at that time (S1 Table) and different child age-related exposures (e.g., younger children may be more likely to consume soil). We were unable to blind study participants due to the nature of the interventions; however, our outcomes were objective indicators of infection analyzed by blinded laboratory technicians, and blinded analysts replicated the data analysis.

During our trial, Kenya implemented a national school-based targeted MDA program to reduce STH prevalence [49], and 43% of study children were reported to have consumed deworming medication in the past 6 months (S8 Table). Reported consumption of deworming medicine was similar across study arms, suggesting no systematic differences in program coverage or intensity between arms (S9 Table). We observed similar *Ascaris* prevalence among study index children (23%, median age 2 years) and older children (22%, median age 5 years), suggesting that school-based MDA could be missing a key reservoir of infection among young, preschool-aged children. Moreover, an environmental survey conducted during the national deworming program in our study region reported common detection of STH eggs in soil collected from the entrance to homes, with *Ascaris* eggs detected in soil in 19% of households [50]. Taken together, these findings suggest that additional control strategies beyond school-based deworming might be necessary to fully interrupt environmental STH transmission.

In contrast to most previous trials evaluating the effect of WSH or nutrition on STH infection, administering deworming medication was not included with our intervention. Our findings represent the potential impact of WSH and nutrition interventions in the context of exposure to a deworming program implemented at the national scale. Although the magnitude of *Ascaris* prevalence reduction observed in the WSH and water arms may be lower than what could be achieved by drug treatment in the short term, reduced STH infection after 2 years of intervention exposure indicates sustained impact. Our results support the proposal that improved WSH could complement chemotherapy in the global effort to eliminate STH transmission.

## Supporting information

S1 Table Indicators of intervention adoption 1 year after intervention delivery began. (DOCX)

S2 Table Indicators of intervention adoption 2 years after intervention delivery began. (DOCX)

S3 Table Characteristics of children included in analysis compared to children lost to follow-up. (DOCX)

S4 Table Characteristics of children included in analysis compared to children lost to follow-up, by treatment status. (DOCX)

S5 Table Unadjusted and adjusted effects of interventions on *Ascaris*, *Ascaris* infection intensity, hookworm, *Trichuris*, STH coinfection, *Giardia*, and STH and *Giardia* coinfection. (DOCX)

S6 Table Estimates of the effect of the combined WSH and combined WSH plus nutrition interventions on child helminth infections by Kato–Katz microscopy and qPCR. (DOCX)

S7 Table Effect of combined WSH intervention compared to single interventions, and effect of combined WSH with nutrition compared to nutrition alone and WSH, on fecal egg count reduction (FECR) with geometric and arithmetic means. (DOCX)

S8 Table *Ascaris* and *Giardia* infection prevalence ratios (PR) by subgroup, compared to control arm. (DOCX)

S9 Table Prevalence of children who reported consumption of deworming medication in the past 6 months by study arm. (DOCX)

S10 Table Quality-control analysis: Comparison of matched sample results by qPCR analyzed in 2 different labs. (DOCX)

S1 Text Additional methods and results. (DOCX)

S2 Text CONSORT checklist. (DOCX)

## Abbreviations

ELISA: enzyme-linked immunosorbent assay
epg: eggs per gram
FECR: fecal egg count reduction
ICC: intraclass correlation coeffcient
LNS: lipid-based nutrient supplement
MDA: mass drug administration
PR: prevalence ratio
qPCR: quantitative polymerase chain reaction
RCT: randomized controlled trial
STH: soil-transmitted helminth
WSH: water treatment, sanitation, and handwashing
WSHN: water treatment, sanitation, handwashing, and nutrition
