# Supplementary material for: Effects of single and integrated water, sanitation, handwashing, and nutrition interventions on child soil-transmitted helminth and Giardia infections: A cluster-randomized controlled trial in rural Kenya
Source: PLoS Med. 2019 Jun 26;16(6):e1002841. doi: 10.1371/journal.pmed.1002841 (PMC6594579; doi:10.1371/journal.pmed.1002841)
Supplement: S2 Table — (DOCX) [file pmed.1002841.s002.docx]

**S2 Table** Indicators of intervention adoption two years after intervention delivery began. Safe child feces disposal was defined as the index child’s last defecation in a latrine, or in a potty (or diaper) and disposed into the latrine. Protected water sources include borewells, protected springs, protected dug wells, rainwater collection and piped water into the home or yard/plot. Note: expected LNS sachet consumption can be >100% because extra sachets were delivered to ensure supplies did not run out.
