# Supplementary material for: Effects of single and integrated water, sanitation, handwashing, and nutrition interventions on child soil-transmitted helminth and Giardia infections: A cluster-randomized controlled trial in rural Kenya
Source: PLoS Med. 2019 Jun 26;16(6):e1002841. doi: 10.1371/journal.pmed.1002841 (PMC6594579; doi:10.1371/journal.pmed.1002841)
Supplement: S5 Table — (DOCX) [file pmed.1002841.s005.docx]

**S5 Table.** Unadjusted and adjusted effects of interventions on *Ascaris*, *Ascaris* infection intensity, hookworm, *Trichuris*, STH co-infection, *Giardia*, and STH and *Giardia* co-infection. Prevalence ratios (PR) estimated with targeted maximum likelihood estimation.
