# Supplementary material for: Effects of single and integrated water, sanitation, handwashing, and nutrition interventions on child soil-transmitted helminth and Giardia infections: A cluster-randomized controlled trial in rural Kenya
Source: PLoS Med. 2019 Jun 26;16(6):e1002841. doi: 10.1371/journal.pmed.1002841 (PMC6594579; doi:10.1371/journal.pmed.1002841)
Supplement: S6 Table — (DOCX) [file pmed.1002841.s006.docx]

**S6 Table.** Estimates of the effect of the combined WSH and combined WSH plus nutrition interventions on child helminth infections by Kato-Katz and qPCR. PR’s estimated by targeted maximum likelihood estimation.

|  | **Kato-Katz** | | **qPCR** | |
| --- | --- | --- | --- | --- |
| ***Ascaris*** | **Prev. (%), total N** | **PR (95% CI)** | **Prev. (%), total N** | **PR (95% CI)** |
| Control | 22.6%, N=2335 |  | 26.8%, N=1036 |  |
| WSH | 17.3%, N=1058 | 0.78 (0.63, 0.96) | 20.9%, N=968 | 0.79 (0.64, 0.97) |
| WSHN | 17.8%, N=1174 | 0.78 (0.63, 0.96) | 21.0%, N=1089 | 0.77 (0.64, 0.93) |
|  |  | |  | |
| ***Trichuris*** |  |  |  |  |
| Control | 1.2%, N=2335 |  | 1.4%, N=1037 |  |
| WSH | 0.5%, N=1058 | 0.44 (0.18, 1.10) | 1.2%, N=968 | 1.24 (0.52, 2.94) |
| WSHN | 0.4%, N=1174 | 0.34 (0.09, 1.31) | 1.0%, N=1089 | 0.67 (0.17, 2.68) |
|  |  | |  | |
| ***Hookworm*** |  |  |  |  |
| Control | 2.2%, N=2335 |  | 6.1%, N=1037 |  |
| WSH | 1.7%, N=1058 | 0.77 (0.45, 1.31) | 7.2%, N=968 | 1.21 (0.78, 1.86) |
| WSHN | 3.0%, N=1174 | 1.34 (0.83, 2.15) | 7.4%, N=1089 | 1.26 (0.77, 2.04) |
