# Supplementary material for: Effects of single and integrated water, sanitation, handwashing, and nutrition interventions on child soil-transmitted helminth and Giardia infections: A cluster-randomized controlled trial in rural Kenya
Source: PLoS Med. 2019 Jun 26;16(6):e1002841. doi: 10.1371/journal.pmed.1002841 (PMC6594579; doi:10.1371/journal.pmed.1002841)
Supplement: S7 Table — (DOCX) [file pmed.1002841.s007.docx]

**S7 Table.** Effect of combined WSH intervention compared to single interventions, and effect of combined WSH with nutrition compared to nutrition alone and WSH, on fecal egg count reduction (FECR) with geometric and arithmetic means. FECRs below are expressed as proportions (percentage change/100). Values of 0.5 epg substituted for samples below the detection limit to calculate log-transformed mean.
