# Supplementary material for: Effects of single and integrated water, sanitation, handwashing, and nutrition interventions on child soil-transmitted helminth and Giardia infections: A cluster-randomized controlled trial in rural Kenya
Source: PLoS Med. 2019 Jun 26;16(6):e1002841. doi: 10.1371/journal.pmed.1002841 (PMC6594579; doi:10.1371/journal.pmed.1002841)
Supplement: S8 Table — (DOCX) [file pmed.1002841.s008.docx]

**S8 Table.** *Ascaris* and *Giardia* infection prevalence ratios (PR) by subgroup, compared to control arm. PR’s estimated by targeted maximum likelihood estimation. Effect modification test p-value estimated by generalized linear models.
