# Supplementary material for: Effects of single and integrated water, sanitation, handwashing, and nutrition interventions on child soil-transmitted helminth and Giardia infections: A cluster-randomized controlled trial in rural Kenya
Source: PLoS Med. 2019 Jun 26;16(6):e1002841. doi: 10.1371/journal.pmed.1002841 (PMC6594579; doi:10.1371/journal.pmed.1002841)
Supplement: S10 Table — (DOCX) [file pmed.1002841.s010.docx]

**S10 Table.** Quality-control analysis: comparison of matched sample results by qPCR analyzed at KEMRI and at Smith College. A total of 379 samples were processed in duplicate by lab technicians at KEMRI and at Smith College. qPCR results from each lab showed excellent concordance. Agreement between the two labs was 97% for *Ascaris* (Kappa-statistic measure of interrater agreement=0.92, p<0.001)*,* 100% for *Trichuris* (Kappa=1.0, p<.0001)*,* and 98% for hookworm (Kappa=0.85, p<0.001). The observed discrepancies could be explained by non-homogenous mixing between stool sample aliquots processed in the separate labs.

|  |  |  |  |
| --- | --- | --- | --- |
| ***Ascaris*** | *KEMRI -* | *KEMRI +* | *Total* |
| *Smith -* | 293 | 8 | **301** |
| *Smith +* | 2 | 76 | **78** |
| *Total* | **295** | **84** | **379** |
|  |  |  |  |
| ***Trichuris*** | *KEMRI -* | *KEMRI +* | *Total* |
| *Smith -* | 377 | 0 | **377** |
| *Smith +* | 0 | 2 | **2** |
| *Total* | **377** | **2** | **379** |

| **Hookworm** | *KEMRI -* | *KEMRI +* | *Total* |
| --- | --- | --- | --- |
| *Smith -* | 346 | 4 | **350** |
| *Smith +* | 4 | 25 | **29** |
| *Total* | **350** | **29** | **379** |
