# Supplementary material for: Effects of single and integrated water, sanitation, handwashing, and nutrition interventions on child soil-transmitted helminth and Giardia infections: A cluster-randomized controlled trial in rural Kenya
Source: PLoS Med. 2019 Jun 26;16(6):e1002841. doi: 10.1371/journal.pmed.1002841 (PMC6594579; doi:10.1371/journal.pmed.1002841)
Supplement: S1 Text — (DOCX) [file pmed.1002841.s011.docx]

**Additional Methods**

*Measurement of intervention adherence*

Adherence to the interventions was measured during unannounced household visits after one year and two years of intervention exposure. During these visits, field staff collected and tested stored drinking water samples for the presence of a chlorine residual, observed latrines to determine the presence of a slab, asked caregivers if their child’s most recent defecation was disposed into the latrine, observed if water and soap was present at a designated handwashing station, and recorded how many sachets of LNS the caregiver reported their child had consumed in the past week.

*Stool sample analysis*

A small number of stool specimens did not contain enough material to form sufficient additional aliquots after Kato-Katz analysis (Figure 1). Aliquots stored for ELISA and qPCR analysis were stored at -80 degrees Celsius until analysis. DNA was extracted from stool samples selected for qPCR assessment (from the control, WSH, and WSHN arms) by the FastDNA spin kit for soil extraction (MP Biomedicals, Santa Ana, California). qPCR assays were run on each sample in duplicate and the two cycle threshold (Ct) values averaged together. In order to ensure the adequate recovery of DNA during the extraction process, thereby reducing the risk of sample false negativity, each stool sample was spiked with 100pg of a previously described internal amplification control (IAC) plasmid. Following extraction, recovery of this plasmid was assessed through the amplificaiton of a unique plasmid-encoded real-time PCR-based target sequence.  Samples which failed to produce a positive signal for IAC underwent re-extraction, and DNA products were again tested for the presence of IAC signal.  Samples for which an internal control assay did not amplify were excluded from the analysis due to the possibility of assay inhibition. A subset of stool samples processed for qPCR was randomly selected for qPCR QA/QC analysis at Smith College. Samples selected for QA/QC were divided into two aliquots, and one aliquot was shipped on dry ice to Smith College for analysis. The same DNA extraction and qPCR analysis protocols were performed at Smith (see Table S9).

*Statistical analysis*

We screened the following covariates for inclusion in the adjusted models: month of measurement, child age (months), child sex, mother’s age (years), mother’s education level (incomplete primary, complete primary, any secondary), household food insecurity (dichotomous category of none vs any), number of children < 18 years in the household, number of individuals living in the compound, distance (in minutes) to the household’s primary water source, housing materials (floor, walls, roof) and household assets, (electricity, radio, television, mobile phone, clock, bicycle, motorcycle, stove, gas cooker, car, and number of cows, goats, dogs, chickens).

**Additional results**

Infection prevalence was also compared between combined and single interventions (WSH compared to W; WSH compared to S; WSH compared to H; WSHN compared to WSH; and WSHN compared to N). There were no significant differences in *Ascaris*, *Giardia,* or hookworm infection prevalence for these comparisons (Table S4)*.* The prevalence of *Trichuris* was lower in the WSH arm compared to the handwashing arm (PR: 0.28, 95% CI 0.11, 0.71), while no other comparisons for *Trichuris* were statistically significant. The prevalence of infection with any STH was lower in the combined WSH arm compared to the sanitation arm (PR: 0.83, 95% CI 0.69, 0.99), but not when compared to the water, handwashing, or WSHN arms; the prevalence of any STH in the nutrition arm was not different from the prevalence in the combined WSHN arm (Table S5).
